# Gastric troubles in Iran: The role of social and economic factors in *Helicobacter pylori* infection

**DOI:** 10.34172/hpp.2023.15

**Published:** 2023-07-10

**Authors:** Hamed Zandian, Telma Zahirian Moghadam, Farhad Pourfarzi, Reza Malekzadeh, Satar Rezaei, Sevda Ghorbani

**Affiliations:** ^1^Centre for Public Health and Wellbeing, School of Health and Social Wellbeing, College of Health, Science and Society, University of the West of England, Bristol, UK; ^2^Social Determinants of Health Research center, Ardabil University of Medical Sciences, Ardabil, Iran; ^3^Digestive Disease Research Center, Ardabil University of Medical Sciences, Ardabil, Iran; ^4^Digestive Oncology Research Center, Digestive Diseases Research Institute, Shariati Hospital, Tehran University of Medical Sciences School of Commerce, Tehran, Iran; ^5^Research Center for Environmental Determinants of Health, Health Institute, Kermanshah University of Medical Sciences, Kermanshah, Iran; ^6^Department of Community Medicine, School of Medicine, Ardabil University of Medical Sciences, Ardabil, Iran

**Keywords:** Disease prevalence, Helicobacter infections, Socioeconomic factors, Stomach neoplasms

## Abstract

**Background:**

*Helicobacter pylori* infection is a major risk factor for gastric cancer in Iran, but the impact of socioeconomic factors on its prevalence is poorly understood. This study aimed to assess the socioeconomic inequalities and risk factors associated with *H. pylori* infection in Iran.

**Methods:**

This cross-sectional study was conducted based on the PERSIAN cohort study. A total of 20460 individuals aged 35 to 70 years in Ardabil, Iran were included in the study. *H. pylori* infection was determined based on stool tests and clinical records. Multilevel logistic regression models with random intercepts at household and community levels were used to identify risk factors associated with *H. pylori* prevalence. The concentration index (CIn) and concentration curve (CC) were employed to assess socioeconomic-related inequality.

**Results:**

In this study, 70.4% (CI 69.6–71.0) of the participants were infected with *H. pylori*, with a higher prevalence in women (71.2%) than men (69.6%). Age (OR: 1.37, CI: 1.17-1.61), sex (OR: 1.20, CI: 1.12-1.28), level of education (OR: 1.33, CI: 1.17-1.49), cardiac disease (OR: 1.32, CI:1.18-1.46), and BMI groups (OR: 2.49, CI: 1.11-5.58) were significantly associated with *H. pylori* infection based on the multivariable logistic regression. The results of the CIn and CC indicated that *H. pylori* were more prevalent among economically disadvantaged groups (CIn: -0.1065; [-0.1374 to -0.0755]).

**Conclusion:**

The prevalence of *H. pylori* in Iran is higher than in other developing countries, and significant socioeconomic inequality exists between the poor and the rich. To reduce the rate of gastric cancer, socio-economic and demographic factors, especially the poor and people with low levels of education, should be considered.

## Introduction

*Helicobacter pylori* infection is now accepted as the major cause of gastritis and has been etiologically linked to duodenal ulcer disease, gastric ulcer disease, and gastric cancer.^1^ Once H. pylori infection is acquired, it seems that the duration of the infection is very long, possibly lifelong.^2^ The prevalence of *H. pylori* infection varies among races, ethnic groups, and between developing and developed countries. However, it is unknown whether this difference is a result of different social and environmental factors or genetic predisposition.^3^ According to previous studies, the prevalence of this infection has been 34.7% and more than 50%, respectively, in developed and developing countries. In Iran, it has varied between 50% and 60%.^4,5^ variation in the prevalence of *H. pylori* infection suggests that cultural background, genetic predisposition, and/or socioeconomic-related environmental factors might play roles in its acquisition and transmission.^6^ Common risk factors for *H. pylori* infection include low socioeconomic status (SES) and low education levels of mothers and family members who are infected.^7^ The prevalence of *H. pylori* has been reported among different populations in terms of SES in different populations.^8^ Childhood is a period of major risk for *H. pylori* infection. Nonetheless, a part of the population becomes infected during adulthood. Researchers have attempted to identify factors that increase the risk of *H. pylori* infection among different societal groups.^9^

 Various studies have shown that the rate of *H. pylori* infection is higher in the Middle East.^10^ In Iran, the rate has varied in various regions of the country based on socioeconomic conditions.^11^ Ardabil province in the northwest of Iran is considered the focal point of gastric adenocarcinoma, where this cancer is known as the most common tumour in the region.^12^ Therefore, assessing the effect of socioeconomic inequality on the prevalence of this microorganism in this region is of great importance. Our aim is to determine whether socioeconomic characteristics are associated with *H. pylori* prevalence, and if so, this will help us explain the factors affecting gastric adenocarcinoma and *H. pylori*.

## Materials and Methods

###  Study setting and sample

 In this cross-sectional study, we investigated the prevalence of *H. pylori* infection and its associated risk factors among adults aged 35-70 years residing in Ardabil, a city in the northwest of Iran.^13^ We utilized data collected from the Ardabil Non-Communicable Disease (ArNCD) cohort study, which is part of the Prospective Epidemiological Research Studies in IrAN (PERSIAN) cohort.^14^ The PERSIAN cohort is a large-scale study conducted across multiple sites in Iran to support the development of healthcare policies for non-communicable diseases. A total of 20 525 individuals were initially enrolled in the ArNCD cohort study between May 2017 and February 2020, with 20 460 participants ultimately using the consensus sampling method included in our analyses after accounting for missing data.^14,15^ The study recruited individuals primarily of Turk ethnicity who were of Iranian origin and spent at least 9 months annually in the study region. Participants with physical or intellectual disabilities that could hinder their ability to complete the admission process were excluded. The study was limited to individuals with Iranian citizenship who resided in Ardabil. Deaf, blind, and individuals with cerebral palsy and those with mental disorders, mental retardation, or any acute psychiatric illness were excluded from the PERSIAN cohort. In addition, based on the PERSIAN cohort protocol, we acknowledge that the use of antibiotics, bismuth, or proton pump inhibitors (PPIs) in the past weeks might affect the test results. Participants who had taken antibiotics, bismuth, or PPIs in the past 4 weeks or 2 weeks were asked to return after 4 weeks of stopping the medication for registration and testing for Helicobacter pylori. In addition, participants with a history of chemotherapy, multicancer treatment, and positive history of eradication therapy were excluded from the study.

###  Data and variables

 The dependent variable of our study was *H. pylori* infection. We used a recent positive stool test (*H. pylori* stool antigen (HpSAg) test) to detect *H. pylori* infection. The HpSAg test is suitable for large-scale population-based studies where cost and ease of implementation are important factors. The cutoff value for the HpSAg test varies depending on the study, the population being tested, and the specific kit used for the test. In general, high sensitivity and specificity is desired to ensure the accurate detection of *H. pylori* infection. One study reported a sensitivity of 96.9% and a specificity of 97.8% using a commercial HpSAg kit with a cutoff value of 20 ng/mL.^16^ However, it is important to note that the optimal cutoff value may vary based on the population being tested and the specific kit used for the test. In this study, we concerned 20 ng/mL as the cutoff value for HpSAg test.

 We also collected information on several independent variables, including age, sex, marital status, education level, body mass index (BMI), occupation, and self-reported non-communicable diseases (i.e., cardiovascular disease, diabetes, and hypertension). To assess SES, we calculated a wealth index based on participants’ self-reported wealth, asset ownership, and housing characteristics, and divided this index into five quintiles ranging from the poorest to the richest groups.

 We used multivariable logistic regression models with random intercepts at household and community levels to identify risk factors associated with *H. pylori* infection. In our study, the household-level variables included the number of household members, household income, occupation, and education level of the head of the household, the infrastructure of the house, the number of bedrooms, the type of house ownership, the number of annual trips, owning a personal car, the price of a personal car, home furniture (dishwasher, washing machine, wardrobe,...), etc. On the other hand, community-level variables included population size, access to healthcare facilities, and the prevalence of *H. pylori* infection in the community. These variables were chosen based on the literature review, PERSIAN cohort protocol, and their potential association with *H. pylori* infection and were included in the multilevel logistic regression model to identify the risk factors associated with the infection.

 The wealth index in this study was created to represent the participant’s SES and was based on their self-reported wealth. To do this, a novel application of principal component analysis was used to calculate household wealth from a set of ownership variables, many of which were binary or categorical.^17^ This approach transformed the ownership variables into a continuous socio-economic status gradient, with five quintiles ranging from the poorest to the richest groups. In addition to the ownership variables, several other variables were also considered, such as housing, personal car, number of rooms, number of family members, household facilities like refrigerator, dishwasher, etc., and the number of books studied. The wealth index was calculated based on assets, homeownership, home area, and car ownership and price.

 Additionally, we used concentration index (CIn) and curve analyses to examine socioeconomic-related inequalities in *H. pylori* infection prevalence. The final sample size included 20 460 individuals of mainly Azari ethnicities. Our findings may have implications for public health efforts aimed at reducing the prevalence of *H. pylori* infection and associated gastric cancer risk in Iran, particularly among disadvantaged socioeconomic groups.^18^

###  Statistical analysis

 Data were analysed using Stata software, version 14.0.0 (Stata Corp, College Station, TX, USA). The result was dichotomized into “with *H. pylori* coded as 1” and “without *H. pylori* coded as 0” based on the recent stool test result. The categorical variables with their respective 95 % confidence intervals (CIs) were described as proportions. For the numerical variables, the standard deviation (SD) and interquartile ranges, the means were calculated as dispersion measures. Poisson regression was used to achieve the crude and adjusted prevalence and their respective 95% CI. We used Poisson regression to model the prevalence of *H. pylori* infection in the study population, which can be thought of as a count of the number of individuals with the infection. The demographic variables and chronic disease status were included in the model to calculate the association and odds ratio (OR) based on multivariate logistic regression. Finally, the CIn and concentration curves (CC) were used to assess the socioeconomic-related inequality in the prevalence of *H. pylori* in the samples included in this study.^19^ CC and CIn were used since they are the standard and most frequently used tools to assess inequalities in the health economics literature. CIn values ​​ranged from -1 to + 1 and negative values ​​indicated that health scores were more concentrated in groups with lower SES and vice versa for positive values. If the CIn was zero, the health outcomes were evenly distributed across populations. The CIn was defined as per following equation:

 (1)

$$CI_{Wagstaff\;}=\frac{CIn}{1-\mu }$$

where *μ* is the mean of the health variable and *CIn* represents the conventional *CIn*.

 The *CIn* for a variable y can be written:

 (2)

$$CIn\;=\;\sum_{K}^{}\left(\frac{\beta_{Κ\bar{Χ\;}}{}_{{}_{Κ}}}{\mu }\right)\;CIn_{Κ\;}+\;GC_{\in \;}/\;\mu$$

Where *CIn* is the overall concentration index, *μ* indicates the mean of *y* (health outcome variable), *x*_k_ represents the mean of *x*_k_ (determinants), *C*_k_ is the concentration index for *x*_k_, and *GCε* denotes the generalized concentration index for *ε*. It should be noted that *CIn* is equal to the weighted sum of the *CIs* of the *k* determinants, where the weight of *x*_k_ is the elasticity of *y* concerning *x*_k_.

 Since the outcome variable of the present study (*H. pylori* infection) was binary, a non-linear estimation was used. The marginal effects of the *βk* based on the logic model were estimated and used to compute the contributions of the explanatory variables.^20^ Below, the linear approximation of the non-linear estimations is given as equation.^3^

 (3)

$$CI\;=\;\sum_{Κ}\left(\frac{\beta_{Κ}^{m}{\;}_{\bar{Χ\;}}{}_{{}_{Κ}}}{\mu }\right)\;CI_{Κ\;}+\;GC{\;}_{\in \;}/\;\mu$$

Where β_k_^m^ is the marginal effect (dy/dx) of each *x*.

## Results

 In this study, the data of 20 460 people 35-70 years old from the Ardabil cohort were included out of which 54.1% were women, and the rest were men. The mean and standard deviation of the age of the subjects were 49.13 ± 8.57 years. At the education level, 31.8% of people were illiterate, wherein in marital status, more than 91% of people were married. In the study population, the prevalence of *H. pylori* was 70.4% (95% CI, 69.6-71.0), with a higher prevalence among men at 71.2% (95% CI, 70.2-72.2) compared to women at 69.6% (95% CI, 68.7-70.5). Most infected people with H. pylori were in the age range of 45 to 50 years. According to the results, with increasing education level, the prevalence of *H. pylori* decreased in men and women where it was estimated to be 63.1% (95% CI 61.1–65.1) for the population with an academic degree and for illiterate people was 71.5% (95% CI 70.3–72.7). The results also showed that the highest prevalence of *H. pylori* was among self-employed people (71.8%, 95% CI 69.2-73.4). Moreover, the prevalence of *H. pylori* in married people (70.5%, 95% CI 69.8–71.2) was higher than in single people (63.9%, 95% CI 58.1–69.4). However, the prevalence of *H. pylori* in people with diabetes (69.6%, 95% CI 67.5–71.6), with hypertension (67.8%, 95% CI 66.2–69.2), and with heart disease (63.5%, 95% CI 60.9–65.9) were less than the overall prevalence and the prevalence of *H. pylori* among obese people (72.2%, 95% CI 71.1–73.2) was higher than the overall prevalence (Table 1).

**Table 1:** Prevalence of *Helicobacter pylori* infection among the Ardabil population by socio-demographic characteristic (n = 20460)

	**Male (n=9377)**		**Female (n=11083)**		**Total **	
	**Total (%)**	**Infected (%)**	**Total (%)**	**Infected (%)**	**Total (%)**	**Infected (%)**
Age categories						
< 40	1567 (16.7)	891 (16.1)	2466 (22.3)	1378 (21.5)	4033 (19.7)	2269 (19.7)
40-44	1382 (14.7)	845 (15.2)	1798 (16.2)	1068 (16.7)	3180 (15.5)	1913 (15.5)
45-49	1940 (20.7)	1172 (21.1)	2098 (18.9)	1274 (19.9)	4038 (19.7)	2446 (19.7)
50-54	1724 (18.4)	1044 (18.8)	1831 (16.5)	1128 (17.6)	3555 (17.4)	2172 (17.4)
55-59	1289 (13.7)	770 (13.9)	1429 (12.9)	815 (12.7)	2718 (13.3)	1585 (13.3)
60-64	932 (9.9)	542 (9.8)	947 (8.5)	491 (7.7)	1879 (9.2)	1033 (9.2)
> 65	543 (5.8)	283 (5.1)	514 (4.6)	248 (3.9)	1057 (5.2)	531 (5.2)
Marital status						
Single	88 (0.9)	50 (0.9)	253 (2.3)	129 (2.0)	341 (1.7)	179 (1.5)
Married	9220 (98.3)	5457 (98.4)	9391 (84.7)	5439 (85.0)	18611 (91.0)	10896 (91.2)
divorced	23 (0.2)	11 (0.2)	1160 (10.5)	652 (10.2)	1183 (5.8)	663 (5.5)
Other	46 (0.5)	29 (0.5)	279 (2.5)	182 (2.8)	325 (1.6)	211 (1.8)
Education						
illiterate	1849 (19.7)	1153 (20.8)	4681 (42.2)	2710 (42.3)	6530 (31.9)	3863 (32.3)
Primary	2071 (22.1)	1282 (23.1)	2534 (22.9)	1548 (24.2)	4605 (22.5)	2830 (23.7)
Tips	1680 (17.9)	1000 (18.0)	1381 (12.5)	824 (12.9)	3061 (15.0)	1824 (15.3)
Diploma	1869 (19.9)	1102 (19.9)	1591 (14.4)	901 (14.1)	3460 (16.9)	2003 (16.8)
Academic	1908 (20.3)	1010 (18.2)	896 (8.1)	419 (6.5)	2804 (13.7)	1429 (12.0)
Chronic disease						
Have Diabetes	969 (10.3)	561 (10.1)	1418 (12.8)	813 (12.7)	2387 (11.7)	1374 (11.5)
Have Hypertension	1461 (15.6)	821 (14.8)	2786 (25.1)	1595 (24.9)	4247 (20.8)	2416 (20.2)
Have Cardiac Ischemic	797 (8.5)	423 (7.6)	942 (8.5)	471 (7.4)	1739 (8.5)	894 (7.5)
Occupation						
Unemployed	2175 (23.2)	1297 (23.3)	4201 (37.9)	2046 (31.9)	6376 (3116)	3343 (27.9)
Official employee	2823 (30.1)	1587 (28.6)	2294 (20.7)	1914 (29.9)	5117 (25.0)	3501 (29.3)
Self-employed	3010 (32.1)	1804 (32.5)	2172 (19.6)	1463 (22.8)	5182 (25.3)	3267 (27.3)
Retired	1369 (14.6)	859 (15.5)	2416 (21.8)	979 (15.3)	3785 (18.5)	1838 (15.4)
BMI					
Underweight	72 (0.8)	38 (0.7)	33 (0.3)	18 (0.3)	105 (0.5)	56 (0.5)
Normal weight	2146 (22.9)	1240 (22.4)	1053 (9.5)	560 (8.7)	3199 (15.6)	1800 (15.1)
Overweight	4575 (48.8)	2691 (48.5)	3895 (35.1)	2221 (34.7)	8470 (41.4)	4912 (41.1)
obesity	2584 (27.6)	1578 (28.4)	6102 (55.1)	3603 (56.3)	8686 (42.5)	5191 (43.4)

BMI, body max index.

**Figure 1:**
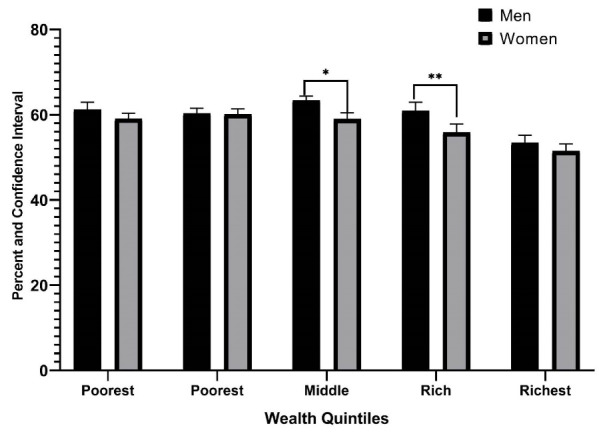
Prevalence of *Helicobacter pylori* infection by gender (male and female) in income quintiles. Based on the results, the prevalence of *H. pylori* infection was higher among men than women, where this difference was significant between men and women in the population with the middle class (*P < 0.05) and the rich(**P < 0.01). Designed by GraphPad Prism 9.3.0 (GraphPad Software, San Diego, California, USA)

 In the present study, we classified the study population into five wealth quintiles from poorest to richest. The results of a comparison of the prevalence of *H. pylori* among wealth quintiles are given in Figure 1 based on gender. There was a significant difference between men and women in the middle and rich quintiles (*P* < 0.05).

 The application of multivariable logistic regression (Table 2) demonstrated associations between *H. pylori* infection and age, sex, level of education, non-communicable disease, and BMI factors. The results of this study showed that *H. pylori* was significantly decreased with age. For example, the probability of *H. pylori* in the oldest age group (65 years and older) was about 1.37 (95% CI, 1.17 to 1.61) times lower than that of the age group under 40 years (reference age group). The results also showed that the pattern of *H. pylori* infection was different concerning gender groups wherein it was 1.20 (95% CI, 1.12 to 1.28) times more likely in males than female to become infected. The prevalence of infection in people with a college education (academic degree) was less than in illiterate people (1.33 [95% CI, 1.17 to 1.49] times less). People with different non-communicable diseases also showed different patterns of *H. pylori* infection. In this regard, people with cardiac disease were significantly infected with *H. pylori*, which was 1.32 (95% CI, 1.18 to 1.46) times higher than healthy people. Nonetheless, there was no difference in terms of *H. pylori* infection between people with diabetes and hypertension with healthy people. On the other hand, the study results showed insignificant differences between different occupation groups in terms of *H. pylori* infection (*P* > 0.05). According to the results of the study, the odds of *H. pylori* infection were significantly increased with elevated BMI wherein the rate of obese people infected with *H. pylori* was 2.49 (95% CI, 1.11 to 5.58) times higher than normal-weight people.

**Table 2:** Multivariable logistic regression model for the association between socio-demographic factors and *Helicobacter pylori* infection among the Ardabil population

	**OR**			
	**Crude (95% CI)**	***P*** ** value**	**Adjusted (95% CI) **	***P *** **value**
Age categories				
< 40 (ref.)	1	-	1	-
40-44	1.17 (1.06 to 1.29)	0.001	1.10 (1.01 to 1.22)	0.034
45-49	1.19 (1.09 to 1.30)	< 0.001	1.10 (1.01 to 1.21)	0.031
50-54	1.22 (1.11 to 1.33)	< 0.001	1.12 (1.01 to 1.24)	0.019
55-59	1.08 (0.98 to 1.20)	0.094	1.00 (0.90 to 1.11)	0.935
60-64	0.94 (0.85 to 1.05)	0.354	0.88 (0.78 to 1.00)	0.057
> 65	0.78 (0.68 to 0.89)	< 0.001	0.73 (0.62 to 0.85)	< 0.001
Gender				
male	1.05 (1.00 to 1.11)	0.044	1.20 (1.12 to 1.28)	< 0.001
Marital status				
Single (ref.)	1	-	1	-
Married	1.27 (1.03 to 1.58)	0.025	1.13 (0.90 to 1.40)	0.274
divorced	1.15 (0.90 to 1.46)	0.245	1.10 (0.85 to 1.42)	0.437
Other	1.67 (1.22 to 2.28)	0.001	1.50 (1.09 to 2.06)	0.011
Education				
Illiterate (ref.)	1	-	1	-
Primary	1.10 (1.01 to 1.18)	0.015	1.02 (0.93 to 1.11)	0.636
Tips	1.01 (0.93 to 1.11)	0.689	0.94 (0.85 to 1.04)	0.273
Diploma	0.94 (0.87 to 1.03)	0.221	0.95 (0.86 to 1.05)	0.374
Academic degree	0.71 (0.65 to 0.78)	< 0.001	0.75 (0.67 to 0.85)	< 0.001
Chronic disease				
Have diabetes	1.03 (0.95 to 1.13)	0.376	0.99 (0.90 to 1.09)	0.915
Have hypertension	1.08 (1.01 to 1.15)	0.024	1.01 (0.94 to 1.09)	0.674
Have cardiac ischemic	1.36 (1.23 to 1.50)	< 0.001	1.32 (1.18 to 1.46)	< 0.001
Occupation				
Unemployed (ref.)	1	-	1	-
Official employee	1.02 (0.97 to 1.16)	0.371	0.99 (0.91 to 1.05)	0.802
Self-employed	0.98 (0.80 to 1.05)	0.114	0.95 (0.83 to 1.02)	0.415
Retired	0.88 (0.68 to 0.93)	0.041	0.84 (0.71 to 0.94)	0.033
BMI				
Normal weight (ref.)	1	-	1	-
Underweight	0.88 (0.60 to 1.31)	0.551	0.85 (0.57 to 1.26)	0.442
Overweight	1.07 (0.98 to 1.16)	0.093	1.11 (1.02 to 1.21)	0.011
obesity	1.14 (1.05 to 1.24)	0.001	1.20 (1.10 to 1.32)	< 0.001
Socioeconomic quintiles				
Poorest (ref.)	1	-	1	-
Poor	1.01 (0.92 to 1.10)	0.73	0.99 (0.91 to 1.09)	0.970
Middle	1.04 (0.95 to 1.14)	0.33	1.02 (0.93 to 1.12)	0.556
Rich	0.94 (0.86 to 1.03)	0.226	0.93 (0.85 to 1.03)	0.210
Richest	0.74 (0.68 to 0.81)	< 0.001	0.78 (0.69 to 0.87)	< 0.001

CI, Confidence Interval; OR, odds ratio; BMI, body max index.

 A significant difference was found between the richest and poorest people in odds of *H. pylori* infection. As per the results, the prevalence of *H. pylori* infection among the richest group was 1.31 (95% CI, 1.08 to 1.72) times lower than the poorest group.

**Figure 2:**
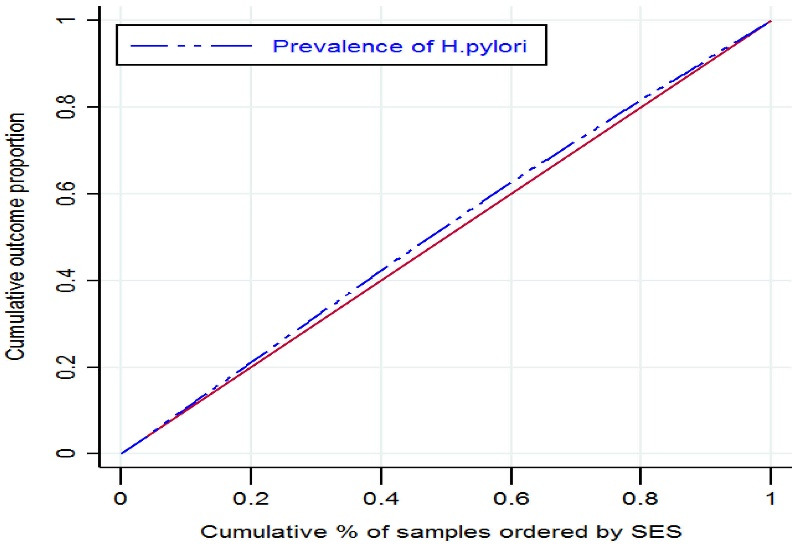
The concentration curve prevalence of *Helicobacter pylori* among adults in ArNCD cohort. Designed by STATA version 14 (StataCorp LLC, College Station, TX)

 The results of the CIn and CC are presented in Table 3 and Figure 2. Based on the results, *H. pylori* was more prevalent among the economically disadvantaged groups (CIn: -0.1065; 95% CI: -0.1374 to -0.0755). Similar findings were found for men (CIn: -0.09914; 95% CI: -0.1321 to -0.0661) and women (CIn: -0.1075; 95% CI: -0.1956 to -0.0194).

**Table 3:** The results of the concentration index for the prevalence of *Helicobacter pylori* in Ardabil Non-Communicable Disease (ArNCD) cohort study

	**Concentration index**	**95% Confidence interval**
Whole of samples	-0.1065	-0.1374 to -0.0755
Males	-0.0991	-0.1321 to -0.0661
Females	-0.1075	-0.1956 to -0.0194

## Discussion

 Out of 20 152 adults from Ardabil, the prevalence of *H. pylori* infection was found to be 70.4% as per the results of this study. However, based on various studies in this field, the prevalence of *H. pylori* varied in African countries: from 40.9% in Egypt to 87.7% in Nigeria; in Latin America: from 49% in Argentina to 74.6% in Chile; in North America: from 35.6% to 41.4%; in Eastern Europe: from 41.2% to 78.5%, and in Western Europe: from 22.1% in Denmark to 82.5% in Estonia. The values in Southeast Asia varied from 28.6% in Malaysia to 70.3% in Vietnam, 24% in Australia and New Zealand, and Western Europe between 18.9 in Switzerland and 46.9% in France. In Turkey, it has been reported to be 77.2%, in India 63.5%, and in Iran.^21^ However, the prevalence of *H. pylori* has been declining in the highly industrialized countries of the western world, while highly prevalent in developing countries.^22^ These differences in *H. pylori* prevalence likely reflect the effects of the level of urbanization,^23^ sanitation, access to clean water,^24^ and SES.^25^

 In the present study, the prevalence of *H. pylori* was higher in men than women. The results were in line with most similar studies in different countries, where in a systematic review conducted by Schröder et al^26^ it was found that male was associated with a greater prevalence of *H. pylori* infection. Ferro et al reported that men had significantly higher odds of infection by *H. pylori*.^27^ However, in a number of studies, women were reported at higher risk of *H. pylori* infection than men.^28^ Different exposures to antibiotics among the sexes have been hypothesized to explain sex differences in Helicobacter pylori infection. In general terms, women are more exposed to the healthcare system,^27^ which can provide them with more opportunities to obtain prescriptions. This is because women are more likely to receive antibiotics than men in their lifetime.^29^*H. pylori* infection and health conditions are found to be inversely related. Although some studies disagree with this hypothesis, the corresponding reasons are still unclear.^30^

 According to this study, the highest prevalence of *H. pylori* was in the age group of 50 to 54 years and then decreased with age. There is disagreement about the relationship between age and prevalence of *H. pylori*. Several studies^29^ have shown that the prevalence of *H. pylori* infection has increased with age and its prevalence was lower in people under 20 years age. Shi et al^31^ showed that the prevalence at people under 20 years old was like that of adults. Chen et al^29^ showed that *H. pylori* infection was significantly different with age (from 5 to 32% for people under 40 and > 70 years, respectively). In contrast, Wang et al^32^ showed that the prevalence of *H. pylori* infection was similar in men and women and did not show any increasing trend with age.^33^

 In a study by Cohen et al,^34^ higher levels of BMI were common in adults infected with *H. pylori*. Suki and colleagues’ study also showed a positive association between *H. pylori* infection and BMI.^35^ Though, there are studies that oppose the association between *H. pylori* infection and obesity.^36^ Our study showed that the chances of developing *H. pylori* were increased in people with high BMI. This could be explained since obese people are more prone to stomach-related diseases due to poor nutrition habits, thus having a higher chance of getting an infection.^37^

 As per the results of the present study, there was a significant relationship between *H. pylori* infection and cardiovascular disease. Similar studies have shown that *H. pylori* infection increased the risk of cardiovascular side effects. *H. pylori* can cause autoimmunity because of molecular mimicry. Therefore, the eradication of *H. pylori* infection as a cardiovascular prevention strategy has been the goal of some studies.^32^

 Given the socioeconomic history of the participants, our data suggested that this factor was also an important risk factor for *H. pylori* infection. This study showed that *H. pylori* infection was higher in the poorest group of the studied population than in the richest and indicated severe odds of *H. pylori* infection due to the poor SES of the participants. Also, the CIn showed that *H. pylori* was more prevalent among the poor groups with a significant inequality in *H. pylori* prevalence. This result is like the results of other studies, indicating a link between low socioeconomic factors and the prevalence of *H. pylori* infection in Asia,^38^ South America^39^ and Africa.^40^ Among the risk factors, the main role can be assigned to poor SES, poor health conditions, overcrowding, and non-compliance with health principles.^41^ This can be concluded that poverty due to poor nutrition, hygiene, and healthcare can increase the transmission rate of *H. pylori* infection.

 On the other hand, the prevalence of *H. pylori* infection was associated with a low level of education and SES.^31^ Therefore, both SES and level of education can be considered as risk factors of *H. pylori* infection. The Genta et al study demonstrated that the prevalence of *H. pylori* was significantly decreased with enhanced income or college education.^42^

 Studies have shown that children with low-educated parents and low SES have been at higher risk of *H. pylori* infection. Severe differences in education levels, especially among mothers, and the prevalence of infection indicated the importance of this element in family health.^43^ Maternal education as a protective factor may act through some approximate determinants that are behavioural in nature. *H. pylori* infection is reducing in many parts of the world as socioeconomic and health conditions improve.

 The present study stands on several merits: application of reliable PERSIAN cohort data, which were obtained through established and controlled methods and having a large sample size. Moreover, the positive and negative results of *H. pylori* incidence were obtained based on laboratory tests in the laboratory of the Gastroenterology Center and analysis-based tools were used to assess the status of *H. pylori*. Iranian society is not homogeneous, and this can be seen in the results of the study well. Herein, data on children and the population under 35 years of age were excluded as children and age groups under 35 years were not included in the PERSIAN cohort sample population. This study had some limitations that must be considered. First, it was conducted exclusively in the northwest region of Iran, so caution should be exercised when generalizing the findings to other populations with different cultures. Although some associations, such as age and *H. pylori* infection, were clearly demonstrated in the study, its cross-sectional nature made it impossible to rule out the possibility of inverse causality. In addition, the data was collected only from individuals aged 35-70 years, which could restrict its applicability to other age groups. This narrow age range may also limit the ability to compare the results with studies that have been conducted on broader age groups. These limitations should be carefully considered when interpreting the findings and designing future research in this area.

## Conclusion

 According to the results of this study, the prevalence of *H. pylori* infection in Iran is still higher than in most other developing and developed countries. In addition, there was significant inequality between the poor and the rich in terms of *H. pylori* infection, where *H. pylori* was more prevalent among the economically disadvantaged groups. Considering the main role of *H. pylori* in gastric cancer and the high prevalence of gastric cancer in this region, it is necessary to consider the findings of the present study by formulating interventions and policies to control and reduce the prevalence of *H. pylori* in this area of the country through variables such as lower age, male gender, low education level, people with underlying chronic disease and obese people with the main emphasis on the poor group of the society. This study provides valuable information to inform public health policies and interventions for the prevention and control of *H. pylori* infection and its associated health consequences.

## Acknowledgements

 We would like to thank the managers and staff of the central office of the PERSIAN Cohort Study, for helping us to conduct this study.

## Competing Interests

 The authors declare that they have no competing interests.

## Ethical Approval

 This study has been approved by the ethical committee of Ardabil University of Medical Sciences (ARUMS) with the code IR.ARUMS.REC.1399.611, where they approved that all methods of this study were performed in accordance with the relevant guidelines and regulations. The Ardabil PERSIAN cohort data was used where all participants signed the informed consent. It should be notice that, based on PERSIAN cohort protocol, we used two routine therapeutic methods *for H. pylori *eradication for participants who were infected by *H. pylori. *

## Funding

 This project was financially supported by national Institute for medical Research Development (NIMAD: 962249) and funded by the Ardabil University of Medical Sciences (ARUMS). The funder had no role in study design, data analysis, decision to publish, or preparation of the manuscript.
